# A novel optical technology based on 690 nm and 850 nm wavelengths to assist needle thoracostomy

**DOI:** 10.1038/s41598-021-81225-4

**Published:** 2021-02-16

**Authors:** Chien-Ching Lee, Chia-Chun Chuang, Chin-Li Lu, Bo-Cheng Lai, Edmund Cheung So, Bor-Shyh Lin

**Affiliations:** 1 Institute of Imaging and Biomedical Photonics, National Chiao Tung University, Tainan, Taiwan; 2 Department of Anesthesiology, An Nan Hospital, China Medical University, Tainan, Taiwan; 3 Department of Medical Sciences Industry, Chang Jung Christian University, Tainan, Taiwan; 4 Graduate Institute of Food Safety, College of Agriculture and Natural Resources, National Chung Hsing University, Taichung, Taiwan

**Keywords:** Biological techniques, Biotechnology, Medical research

## Abstract

The sensitivity of pneumothorax diagnosis via handheld ultrasound is low, and there is no equipment suitable for use with life-threatening tension pneumothorax in a prehospital setting. This study proposes a novel technology involving optical fibers and near-infrared spectroscopy to assist in needle thoracostomy decompression. The proposed system via the optical fibers emitted dual wavelengths of 690 and 850 nm, allowing distinction among different layers of tissue in vivo. The fundamental principle is the modified Beer–Lambert law (MBLL) which is the basis of near-infrared tissue spectroscopy. Changes in optical density corresponding to different wavelengths (690 and 850 nm) and hemoglobin parameters (levels of Hb and HbO_2_) were examined. The Kruskal–Wallis H test was used to compare the differences in parameter estimates among tissue layers; all p-values were < 0.001 relevant to 690 nm and 850 nm. In comparisons of Hb and HbO_2_ levels relative to those observed in the vein and artery, all p-values were also < 0.001. This study proposes a new optical probe to assist needle thoracostomy in a swine model. Different types of tissue can be identified by changes in optical density and hemoglobin parameters. The aid of the proposed system may yield fewer complications and a higher success rate in needle thoracostomy procedures.

## Introduction

Needle thoracostomy (NT), a procedure used in chest decompression, is a process in which an angiocatheter is inserted through the chest wall into the pleural cavity to release an abnormal and potentially life-threatening volume of air trapped therein. Tension pneumothorax is a condition that can displace mediastinal structures and compromise cardiorespiratory function, resulting in an insufficient supply of oxygen and detrimental changes to blood pressure. An increase in thoracic intrapleural pressure, which occurs within a relatively non-distensible cavity, can compress the superior vena cava, right atrium, and inferior vena cava, as the pressure in each of these structures is relatively low in the thoracic cavity. Therefore, venous return is impeded and cardiac output is impaired. Immediate chest decompression is required and may be lifesaving. NT is recommended by advanced trauma life support and is traditionally performed in the second intercostal space at the midclavicular line with a 5-cm angiocatheter to decompress the pleural space and rapidly increase venous return to the right atrium^1^. However, the efficacy of this procedure is controversial^2^.

Several reasons have been proposed for the high failure rate of NT with regard to releasing life-threatening tension in pneumothorax conditions. First, the small size, flexibility, and kinking tendency of an intravenous angiocatheter are associated with an increased risk of mechanical failure, resulting in inadequate pneumothorax tension relief. In fact, Martin et al. have suggested that new NT equipment should be designed and current guidelines should be revised^3^. Damages to surrounding tissues, including missed iatrogenic pneumothorax, hemothorax, and intrathoracic organ injury, have been observed as complications associated with the advancement of angiocatheter needles into the pleural space^4,5^. Although a handheld ultrasound is used with an increasing frequency, its sensitivity for diagnosing pneumothorax is very low^6^. Additionally, identifying the location of an angiocatheter needle tip is challenging when using a narrow ultrasound probe that also captures bone shadows and air in the pleural cavity; this process becomes even more complex as the needle penetrates deeper into the tissue. Capnography is one of good tools in detecting tension pneumothorax when the venous return is impeded. In low cardiac output conditions (e.g. cardiac arrest, pulmonary embolism, tension pnumothorax), end-tidal CO_2_ (ETCO_2_) will decrease. In tension pneumothorax condition, needle thoracostomy with colorimetric capnography device are used in some studies^7,8^.

**Figure 1:**
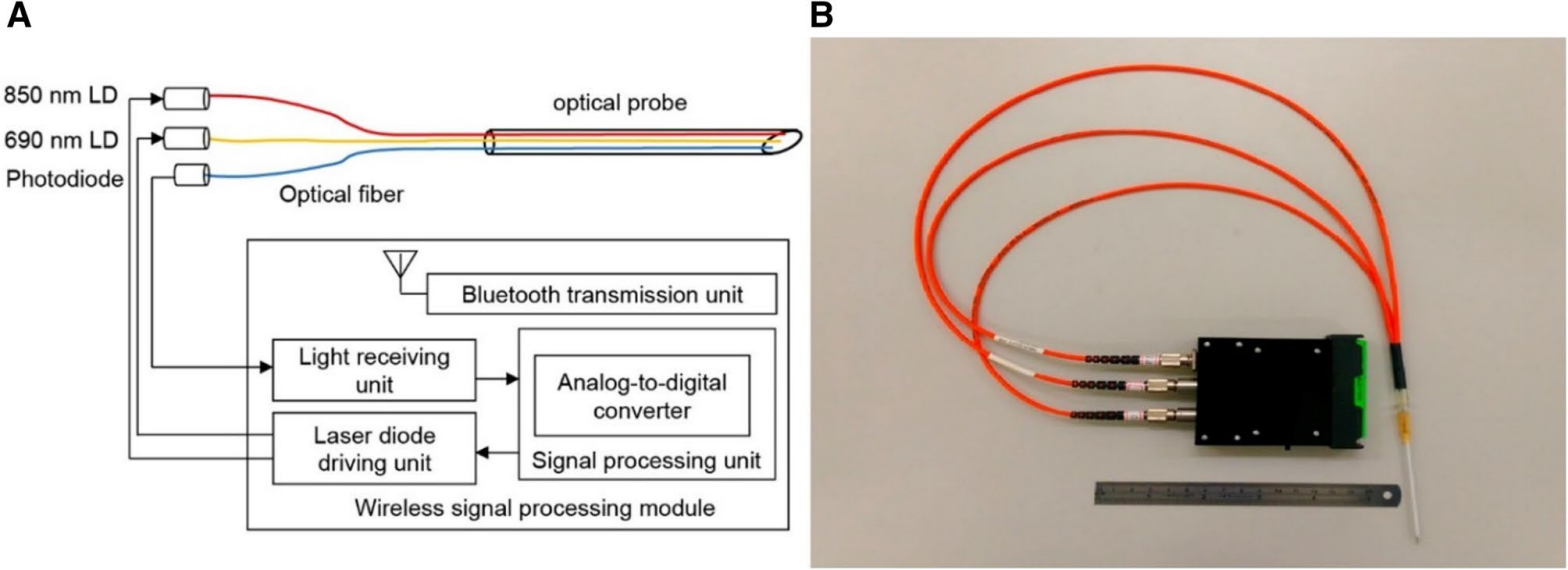
(**A**) Basic structure. (**B**) Photograph of pleural cavity recognition system.

Presently, there is limited equipment for NT. To address this gap, a unique optical pleural space recognition system was proposed using an angiocatheter needle containing dual-wavelength optical fibers to identify different tissue layers. This novel system was based on near-infrared spectroscopy and a dual-wavelength (690 nm and 850 nm) laser light source. The red light and near-infrared light (690 nm and 850 nm, respectively) were emitted from optical fibers consisting of 14-gauge angiocatheter (Fig. 1A,B). When operators advanced angiocatheters into the pleural space, the reflected and scattered light signals were collected and analyzed with specialized software, showing distinct optical properties corresponding to each layer of tissue.

## Results

**Figure 2:**
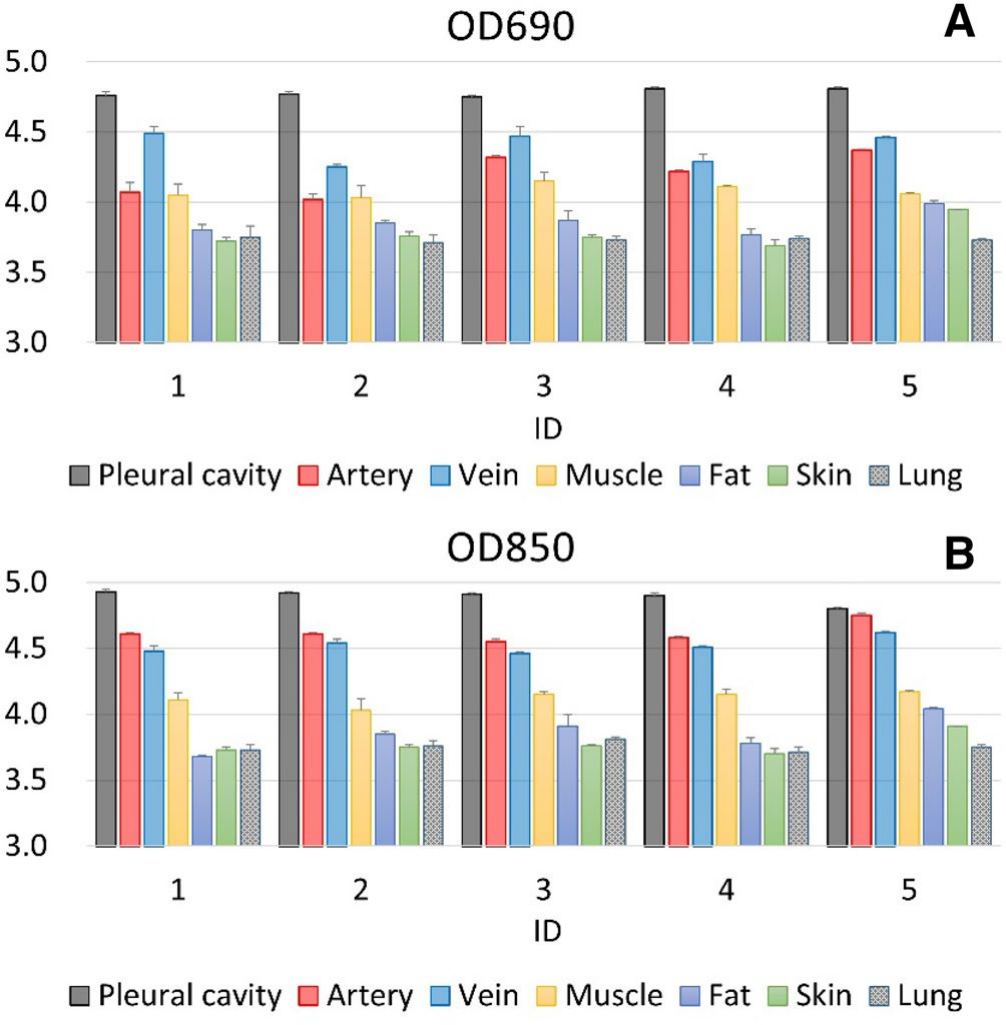
(**A**) 690-nm optical density change and (**B**) 850-nm optical density change in different tissue layers.

The optical intensities associated with the wavelengths of interest in each layer of tissue are presented in Fig. 2. Observed changes in optical density (OD) at both wavelengths were the highest in the pleural cavity, followed by the artery and vein tissues, with relatively lower changes observed in the skin, fat, and lung tissues. In the vein tissues, changes in optical density observed at 690 nm were higher than those observed in the artery. The opposite was true with respect to the 850-nm wavelength; changes in optical density observed at 850 nm were lower in the vein than in the artery. Changes in optical densities (OD) at 690 nm observed in the lung, skin, muscle, and fat tissues were similar to those observed at 850 nm. The Kruskal–Wallis H test was used for statistical analyses of the light signal data of every tissue layer. The fixed effect of tissue was examined in a linear mixed model considering repeated measures as a random effect. For the change in optical density, the pleural cavity was set as the reference category. Statistical significance was reflected in the results of our study. All p-values were < 0.001 relevant to both 690-nm and 850-nm wavelengths.

**Figure 3:**
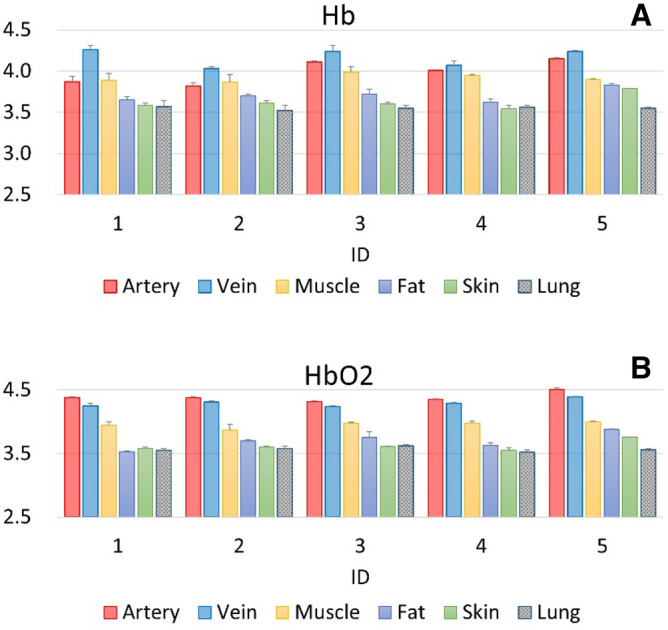
(**A**) Hb concentrations in different tissue layers. (**B**) HbO_2_ concentrations in different tissue layers.

Estimated relative concentrations of Hb and HbO_2_ are presented in Fig. 3. Among all tissue layers, the relative Hb concentration was highest in the vein. However, the relative HbO_2_ concentration was higher in the artery than in the vein, and concentrations of both parameters were higher in the vein and in the artery than in other tissue layers. Nonetheless, relative concentrations of both Hb and HbO_2_ were lower in skin, fat, and lung tissues than in other tissue layers. The Hb and HbO_2_ concentrations were derived from the Eq. (3) according to the modified Beer–Lambert law (MBLL). In Fig. 3, the Kruskal–Wallis H test was also used for statistical analyses of every tissue layer. The fixed effect of tissue was examined in a linear mixed model considering repeated measures as a random effect. For concentrations of Hb, the vein was set as the reference category. For concentrations of HbO_2_, the artery was set as the reference category. Statistical significance was reflected in the results of our study. All p-values were < 0.001 relevant to both the Hb and HbO_2_ concentrations.

### Comparison between the proposed system and other methods

Comparisons between the presented system and other commercially available products are presented in Table 1. Video-assisted thoracoscopic surgery is a minimally invasive surgical technique used to diagnose and treat chest problems^9,10^. During this procedure, the surgeon inserts a thoracoscope with a tiny camera, alongside other surgical instruments, into the patient’s thorax through one or more small incisions in the thoracic wall. Images transmitted by the thoracoscope can help guide surgeons in this process. However, this procedure is cost- and resource-intensive, and it requires an appropriately equipped operating room. Recently, ultrasonography has been used for bedside diagnosis of pneumothorax. In some trauma centers, extended focused assessment with sonography in trauma (FAST) has been used for the rapid bedside examination of thoracic or abdominal organs following trauma^11,12^. Some sonographic features are used in the diagnosis of pneumothorax, including the absence of comet-tail artifact (high sensitivity, low specificity), the absence of lung sliding (high sensitivity and specificity), and the presence of lung point (high specificity, lower sensitivity). The combination of lower specificity in the absence of comet-tail artifact and lower sensitivity in the presence of lung points can be confusing. Finger thoracostomy is a widely accepted procedure which the surgeon creates an incision with a scalpel blade and a curved Kelly into pleural cavity to decompress fluid or air^13^. It must be very careful to prevent finger laceration especially when one patient has multiple rib fractures. It may be potentially increased risk of infection when the glove is broken by the rib fracture. Finger thoracostomy procedure can be done either alone or combined with needle decompression. A computed tomography (CT) scan of the chest is more sensitive for the evaluation and diagnosis of trauma patients with occult pneumothorax^14,15^. In fact, a chest CT is the “gold standard” for detecting occult traumatic pneumothorax, which is difficult to detect on a supine chest radiograph. However, it is not possible to use this method in prehospital emergency care or in areas where access to medical equipment is restricted. The system proposed in the present study has several advantages, including small size (11 × 7.5 × 2.5 cm^3^), convenient setup, low cost, and the capacity to distinguish tissue types at each layer. Overall, this proposed system might be useful in prehospital emergency care or in areas with restricted access to medical equipment.

**Table 1:** Comparison between the proposed system and other methods.

Approaches	The proposed system	Video-assisted thoracoscopic surgery (VATS)	Ultrasonography	Finger thoracostomy	Chest computed tomography
Sensing technology	Near infrared spectroscopy	Video	Ultrasound	Finger	Computed tomography
Transmission mode	Bluetooth	Cable	Cable	–	–
Setup convenience	High	Low	Average	High	Low
System size	Small	Huge	Average	–	Huge
Physiological parameters	Optical Density change, hemoglobin concentration,	3-D image	2-D image	–	2-D and 3-D image
Cost	Low	High	High	Low	High
Advantages	Distinguishability of every tissue layer by layer	Full inspection chest tissues and management	Non invasive and non ionizing radiation	Widely accepted method of pleural decompression	Gold standard for detecting pneumothorax
Applications	New equipment for needle thoracostomy	Surgical intervention	Image study	Surgical intervention	Image study
Limitations	Low image resolution	Huge size and high cost	Low sensitivity and low specificity	Potentially increased risk of infection	Huge size and high cost

## Discussion

NT may be the quickest method to decompress tension pneumothorax in a prehospital setting^16^; however, there is currently a lack of suitable equipment that can conveniently be used in the field. This study proposed a novel system that may help emergency medical service (EMS) teams to provide required intervention without the risk of tissue trauma. Operators can perform chest decompression with a standard chest tube, a pigtail catheter, or an angiocatheter. Most commonly, NT is performed to release thoracic tension prior to the placement of large chest tubes in a prehospital setting or during acute trauma resuscitation. The proposed system may supply EMS teams with an instrument to perform needle decompression in areas with restricted access to medical equipment; it is associated with low cost, convenient setup, and a small size. The proposed pleural cavity recognition system (Fig. 1) consists of the wireless signal processing module (black part; 11 × 7.5 × 2.5 cm^3^) and three orange cables (about 40 cm in length) with optical fibers embedded into the hollow 14-gauge angiocatheter. This system can effectively distinguish between tissue layers, thus allowing EMS teams to perform needle decompression by guiding needle penetration through tissue until the pleural cavity is reached.

Observed changes in optical density (OD) (Fig. 2) at both wavelengths were highest in the pleural cavity and a total 50 times measurements were done in each layer of tissue. At 690 nm, the average changes in optical density (OD) were 4.78 (4.75 ~ 4.81) in five pigs (ID 1 to ID 5). When the needle tip was advanced into the pleural cavity, observed changes in optical density (OD) were dramatically increasing compared with surrounding tissues. At 690 nm, our experimental work justified the result that the needle tip was in the pleural cavity when changes in optical density were around 4.75 ~ 4.81. At 850 nm, the average changes in optical density (OD) were 4.89 (4.80 ~ 4.93) in five pigs (ID 1 to ID 5). When the needle tip was advanced into the pleural cavity, observed changes in optical density (OD) were also dramatically increasing compared with other tissues. At 850 nm, our experimental work justified the result that the needle tip was in the pleural cavity when changes in optical densities were around 4.80 ~ 4.93. We could easily navigate the needle tip into the pleural cavity with the aid of our proposed system.

The conventional NT procedure is the needle inserted along the superior margin of the second or third intercostal space at the midclavicular line^17,18^. However, in recent studies, needles as long as 8 cm have been required to perform this technique effectively^19–21^. Longer angiocatheters may increase the success rate of chest decompression but it may increase risks of unwanted damage. Women have been reported to have significantly thicker chest walls, conferring an increased risk of failure^22^. Meanwhile, a lateral approach to NT is controversial. A previous study reported the distance at the second intercostal space along the midclavicular line to be smaller than that at the fourth intercostal space along the midaxillary line (p < 0.01)^23^. Catheters placed at the lateral site may be dislodged by the patient's arm and may be more prone to obstruction^24^. Hence, angiocatheter placement at the fourth/fifth ICS-AAL may be easier to perform in prehospital settings, and its predicted failure rate of needle decompression is lower^25–27^. New evidence (Table 2) supports the approach from the fourth/fifth ICS-AAL, as it is associated with a lower failure rate than the second ICS-MCL or the fourth/fifth ICS-MAL. Furthermore, advantages of the fourth/fifth ICS-AAL approach include a thinner chest wall and the absence of major blood vessels nearby (e.g. the subclavian vessels and the internal mammary artery)^25^.

**Table 2:** Needle thoracostomy failure rate (modified from Laan et al., 2016)^25^.

Location	Chest wall thickness (cm)	Failure rate with 5 cm angiocatheter
2nd ICS-MCL	4.3 (3.9–4.7)	38% (24–54%)
4th/5th ICS-MAL	4.0 (2.9–5.1)	31% (10–64%)
4th/5th ICS-AAL	3.4 (2.8–4.0)	13% (8–22%)

In our experiment, NT was performed following the conventional protocol; however, angiocatheters were embedded with optical fibers rather than with 5- or 10-mL syringes. Changes in optical densities per tissue type corresponding with both 690-nm and 850-nm wavelengths are presented in Fig. 2, with relative Hb and HbO_2_ concentrations presented in Fig. 3. Optical density changes associated with 690-nm and 850-nm wavelengths were lowest in the pleural cavity, where limited photons were detected by the photodiode. Within artery and vein tissues, the molar extinction coefficient was different from that in other tissues, resulting in a higher rate of light absorption by hemoglobin than that in other tissues; thus, less light was reflected or scattered within these tissues, and the observed changes in optical densities were higher^28–31^. These properties ensured easy differentiation of artery and vein tissues from other tissue layers, including those from skin, fat, and muscle. Within the skin and fat layers, changes in optical density were relatively low due to the low rate of light absorption and the high rate of light reflection. Concurrently, findings from hemoglobin concentration calculations (Fig. 3) were consistent with those from our system of differentiating among tissue layers. Differences in optical properties between tissue types corresponded to differences in hemoglobin concentration; these concentrations were highest in artery and vein tissues, followed by lung and muscle tissues and then by skin and fat tissues. These differences in the optical properties of tissue types can be used to assist operators during NT, helping to prevent unwanted tissue trauma. In our proposed system, there were some limitations. First, subcutaneous emphysema or subcutaneous hematoma may create false positives because it may interfere the signals collection. We intently advanced the needle tip to avoid from subcutaneous emphysema or subcutaneous hematoma. Nevertheless, we couldn’t entirely prevent these situations. Second, there were two seconds lag-time to analyze these collected signals. The lag-time may result in unwanted injuries. This issue can be resolved by a simplified algorithm and an upgraded proposed system in the future.

## Methods

To distinguish among tissue components reached by the needle tip, the proposed optical probe was designed as a needle-type structure enabling tissue penetration (Fig. 1). The proposed system contained an optical probe and a wireless signal-processing module. The optical probe consisted of an angiocatheter, two laser diodes, a photodiode, and optical fibers. The optical fibers were inserted into the angiocatheter (14 G × 2^1/2^ 64 mm, Surflo Angiocatheter) and connected to the laser diodes and the photodiode. The laser diodes were used as a dual-wavelength light source; the photodiode was used to receive the light signal penetrating through the tissue. The wireless signal-processing module consisted of a laser-diode-driving unit, a light-receiving unit, a signal-processing unit, and a Bluetooth transmission unit.

The laser-diode-driving unit was designed to trigger laser diodes to provide light sources at wavelengths of 690 nm and 850 nm. When light was emitted, it was transmitted through the optical fiber to the tip of angiocatheter, illuminating the tissue. Within the tissue, photons were absorbed, scattered, and reflected. Different tissue types have distinct optical properties in response to particular light wavelengths; tissues from the skin, fat, muscle, artery, vein, lung, and pleural cavity absorb, scatter, and reflect photons differently, therefore allowing us to distinguish among tissue types.

Tissue-penetrating light was received by the photodiode and was amplified and filtered by the light-receiving unit of the module. Subsequently, the received light signal was digitized by the signal-processing unit and transmitted via Bluetooth to the processing computer, where the relative deoxy-hemoglobin (Hb) and oxy-hemoglobin (HbO_2_) concentrations were estimated based on the modified Beer–Lambert law; it has been previously reported that optical attenuation in a highly scattering medium, such as human tissue, can be described by this modified Beer–Lambert law^32,33^. The software used for signal analysis was developed by ourselves based on Microsoft Visual C#, 2017, Microsoft, USA. Once the dual-wavelength light beams penetrated through the tissue, they were then absorbed, scattered, reflected, and received by the photodiode of the proposed system. The optical density variation ∆OD was expressed as

1 $$\Delta \mathrm{OD}\left(\lambda \right)=-\mathrm{log}\frac{{I}_{o}\left(\lambda \right)}{{I}_{i}\left(\lambda \right)}=\varepsilon CLB\left(\lambda \right),$$

where $\varepsilon$ was the molar extinction coefficient, $C$ was the molar concentration, $L$ was the distance between the light source and the detector, and ${I}_{o}\left(\lambda \right)$ and ${I}_{i}\left(\lambda \right)$ denoted the light densities of the incident and penetrating light, respectively. The last one parameter $B\left(\lambda \right)$ was the differential path length factor according to the wavelength $\lambda$. $\lambda$ was applied to adjust the path length from light source to detector, and could be expressed as

2 $$\mathrm{B}\left(\lambda \right)=\frac{{C}_{1}^{empirical}}{1+\mathrm{ln}\left(1+L{C}_{2}^{empirical}{\mu }_{a,\lambda }\right)},$$

where the parameters of ${\mu }_{a,\lambda }$ was indicated the absorbing coefficient according to the wavelength $\lambda$. The experience parameter values of ${C}_{1}^{empirical}$ and ${C}_{2}^{empirical}$ were defined as 1.08. and 0.71, respectively^34^.

At wavelengths of 690 nm (red light) and 850 nm (near-infrared light), hemoglobin constituted the main photon absorber in every tissue type. Consequently, for 690-nm and 850-nm light beams, the change in optical density was expressed as

3 $$\Delta \mathrm{OD}\left(\lambda \right)=\left[{\varepsilon }_{Hb{O}_{2}}(\lambda )\times \left[Hb{O}_{2}\right]+{\varepsilon }_{Hb}(\lambda )\times \left[Hb\right]\right]LB\left(\lambda \right),$$

where [HbO_2_] was the relative concentration of oxy-hemoglobin (HbO_2_), [Hb] was the concentration of deoxy-hemoglobin (Hb), and ${\varepsilon }_{Hb{O}_{2}}(\lambda )$ and ${\varepsilon }_{Hb}(\lambda )$ were the molar attenuation coefficients of HbO_2_ and Hb at the wavelength λ, respectively.

Corresponding to the discrepancy in the molar attenuation coefficient (the different absorbing effect) of Hb and HbO_2_, the Hb and HbO_2_ concentrations could be calculated according to the change of optical density corresponding to dual wavelength 690 nm and 850 nm. For these two different light wavelengths of λ_1_ and λ_2_, the Hb and HbO_2_ concentrations could be re-expressed as the Eqs. (4) and (5)

4 $$\left[Hb\right]=\left({\varepsilon }_{Hb{O}_{2}}({\lambda }_{1})\times \frac{\Delta \mathrm{OD}({\lambda }_{2})}{B({\lambda }_{2})}-{\varepsilon }_{Hb{O}_{2}}({\lambda }_{2})\times \frac{\Delta \mathrm{OD}({\lambda }_{1})}{B({\lambda }_{1})}\right)\times \frac{1}{\mathrm{det}(A)}\times \frac{1}{L},$$

5 $$\left[Hb{O}_{2}\right]=\left({\varepsilon }_{Hb}({\lambda }_{2})\times \frac{\Delta \mathrm{OD}({\lambda }_{1})}{B({\lambda }_{1})}-{\varepsilon }_{Hb}({\lambda }_{1})\times \frac{\Delta \mathrm{OD}({\lambda }_{2})}{B({\lambda }_{2})}\right)\times \frac{1}{\mathrm{det}(A)}\times \frac{1}{L}.$$

For light at a near-infrared (NIR) wavelength of about 600–900 nm, hemoglobin is one of the main absorbers^34,35^. The molar extinction coefficients of HbO_2_ and Hb are distinct at 690-nm and 850-nm wavelengths, respectively. Moreover, the isosbestic point of the absorbing spectra of both parameters is approximately 800 nm. To estimate relative concentrations of HbO_2_ and Hb in this study, a 690-nm wavelength laser diode (HL6738MG, Thorlabs, USA) and an 850-nm wavelength laser diode (L850P030, Thorlabs, USA) were used as a dual-wavelength light source. A photodiode (PD15-22C, Everlight, Taiwan) was used as a light detector. These optical parameters, including the optical density variation ∆OD, and HbO_2_ and Hb levels could provide operators with beneficial real-time information during an NT.

### Experiment design

All methods were carried out in accordance with relevant guidelines and regulations. The study was carried out in compliance with the ARRIVE guidelines. The chairman of the Institutional Animal Care and Use Committee Chi Mei Medical Center, Tainan, Taiwan (J.J. Wang), authorized and approved the present study animal use protocol (approval number 106042701). Five pigs, each weighing approximately 25 kg, were used for this in vivo study. General anesthesia with endotracheal tube intubation was performed, and the pigs were maintained and ventilated; an anesthetic drug (isoflurane) was supplied for inhalation. In the first part of the experiment, pigs were dissected and tissue layers (including skin, fat, muscle, pleural cavity, artery, vein, and lung tissues) were separated. The fifth intercostal space-anterior axillary line (ICS-AAL) was chosen for performing an NT. We used 6.4-cm angiocatheters with embedded dual-wavelength optical fibers to collect real-time layer-by-layer data on an oscilloscope. All data were analyzed immediately using specialized software.

**Figure 4:**
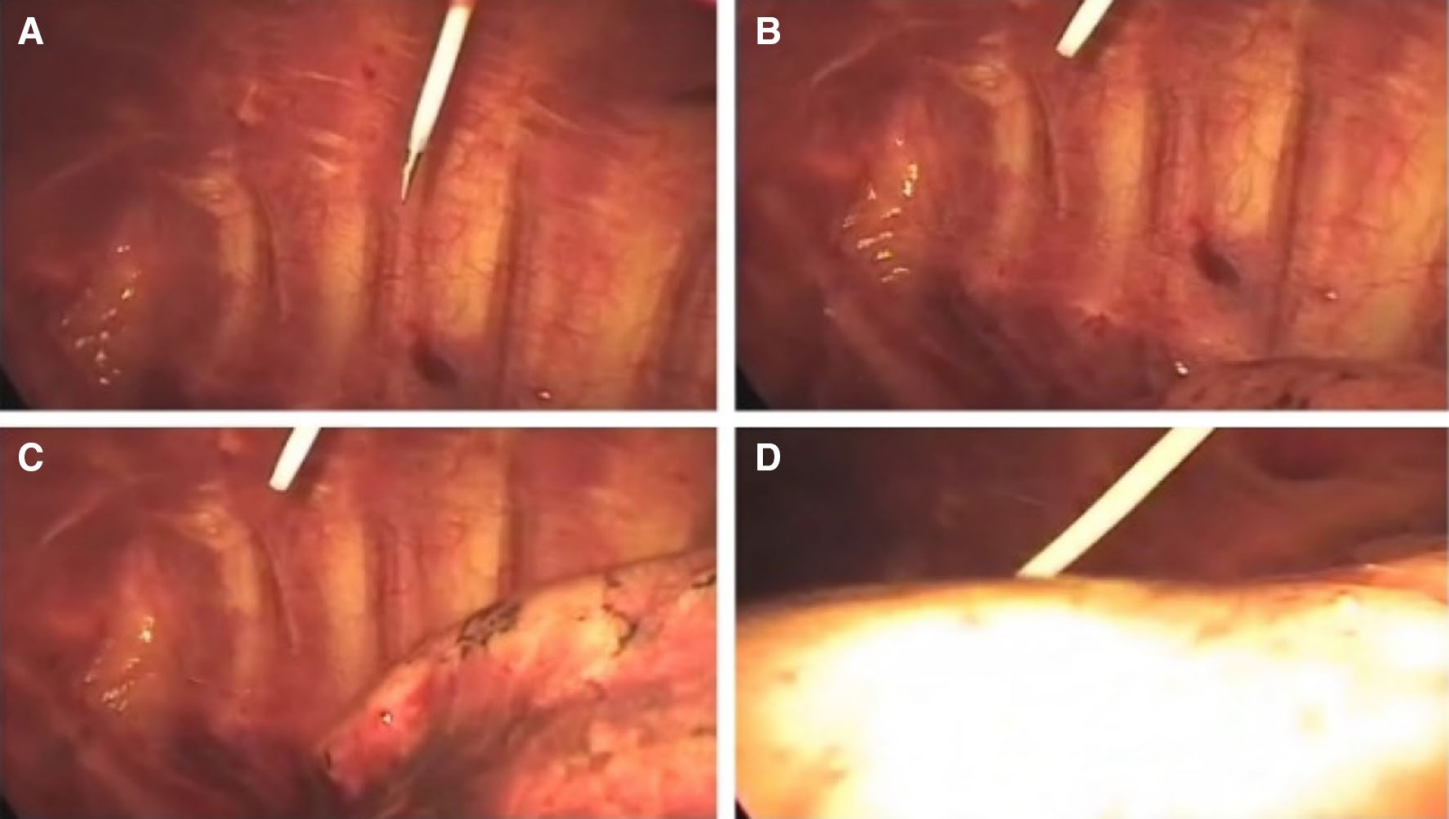
Sequence of needle decompression under the thoracoscope in the second part of the experiment: (**A**) The angiocatheter embedded with optical fibers was inserted into the iatrogenic pneumothorax with the aid of our proposed system. (**B**) The inside introducer needle embedded with optical fibers was removed and the outside plastic sheath was left in the pleural cavity. (**C**) Beginning of lung re-expansion after the iatrogenic pneumothorax. (**D**) Near-full re-expanded lung after needle decompression.

The needle tip embedded with dual-wavelength fibers was inserted into every tissue during NT, and it remained in each tissue layer for approximately 4–5 s to collect data. In the second part of the experiment, we advanced the needle embedded with dual-wavelength optical fibers layer by layer into the iatrogenic pneumothorax under the thoracoscope, aided by the data acquired in the first part of the experiment (Fig. 4A). We removed the inside introducer needle embedded with optical fibers, leaving the outside sheath (Fig. 4B). Gradual re-expansion of the lung was observed after needle decompression (Fig. 4C,D).

### Statistical methods

In an attempt to offset the small sample size (n = 5), we measured changes to the optical density and HbO_2_ and Hb concentrations at wavelengths of 690 nm and 850 nm a total of 10 times per tissue type in each pig. We performed the Kruskal–Wallis H test to compare estimates among all tissue layers. To further examine pairwise differences between tissue layers, we performed a general linear mixed-model regression analysis, which simultaneously considered the random effects of repeated measures and the fixed effects of tissue layers on parameter estimates with the vein (Fig. 3A), and artery (Fig. 3B) sets as reference categories. All statistical analyses in this study were performed using SAS/STAT software, Version 9.4 of the SAS System for Windows. Copyright © 2002–2012 by SAS Institute Inc., Cary, NC, USA.

## Conclusions

The proposed system involves assistance via a needle embedded with optical fibers. Dual-wavelength light beams may help operators differentiate among tissue types and navigate the needle tip into the pleural cavity, supporting smoother delivery of NT. The method proposed in this study may help reduce the rate of complications associated with NT and improve the associated success rate.

## References

[1] Surgeons., A. C. *Advanced Trauma Life Support_: Student Course Manual*. Vol. 9 (2012).

[2] Fitzgerald M, Mackenzie CF, Marasco S, Hoyle R, Kossmann T (2008). Pleural decompression and drainage during trauma reception and resuscitation. Injury.

[3] Martin M, Satterly S, Inaba K, Blair K (2012). Does needle thoracostomy provide adequate and effective decompression of tension pneumothorax?. J. Trauma Acute Care Surg..

[4] Butler KL, Best IM, Weaver WL, Bumpers HL (2003). Pulmonary artery injury and cardiac tamponade after needle decompression of a suspected tension pneumothorax. J. Trauma.

[5] Wernick B (2015). Complications of needle thoracostomy: A comprehensive clinical review. Int. J. Crit. Illn. Inj. Sci..

[6] Oliver P, Bannister P, Bootland D, Lyon RM (2019). Diagnostic performance of prehospital ultrasound diagnosis for traumatic pneumothorax by a UK Helicopter Emergency Medical Service. Eur. J. Emerg. Med..

[7] Zietlow J (2020). Decompression of tension pneumothorax in a trauma patient-first use of a novel decompression colorimetric capnography device in human patient. Gen. Thorac. Cardiovasc. Surg..

[8] Naik ND (2017). Needle decompression of tension pneumothorax with colorimetric capnography. Chest.

[9] Solaini L (2008). Video-assisted thoracic surgery (VATS) of the lung: Analysis of intraoperative and postoperative complications over 15 years and review of the literature. Surg. Endosc..

[10] Imperatori A (2008). Peri-operative complications of video-assisted thoracoscopic surgery (VATS). Int. J. Surg..

[11] Zhang M (2006). Rapid detection of pneumothorax by ultrasonography in patients with multiple trauma. Crit. Care.

[12] Soldati G, Iacconi P (2001). The validity of the use of ultrasonography in the diagnosis of spontaneous and traumatic pneumothorax. J. Trauma.

[13] Jodie P, Kerstin H (2017). BET 2: Pre-hospital finger thoracostomy in patients with chest trauma. Emerg. Med. J..

[14] de Moya MA (2007). Occult pneumothorax in trauma patients: Development of an objective scoring system. J. Trauma.

[15] Neff MA, Monk JS, Peters K, Nikhilesh A (2000). Detection of occult pneumothoraces on abdominal computed tomographic scans in trauma patients. J. Trauma.

[16] Weichenthal L, Crane D, Rond L (2016). Needle thoracostomy in the prehospital setting: A retrospective observational study. Prehosp. Emerg. Care.

[17] Wax DB, Leibowitz AB (2007). Radiologic assessment of potential sites for needle decompression of a tension pneumothorax. Anesth. Analg..

[18] Cotte J, Cungi PJ, Lacroix G, Cungi PJ, Lacroix G (2013). Needle thoracostomy for tension pneumothorax. J. Trauma Acute Care Surg..

[19] Zengerink I (2008). Needle thoracostomy in the treatment of a tension pneumothorax in trauma patients: What size needle?. J. Trauma.

[20] Aho JM (2016). Needle thoracostomy: Clinical effectiveness is improved using a longer angiocatheter. J. Trauma Acute Care Surg..

[21] Clemency BM (2015). Sufficient catheter length for pneumothorax needle decompression: A meta-analysis. Prehosp. Disaster Med..

[22] Givens ML, Ayotte K, Manifold C (2004). Needle thoracostomy: Implications of computed tomography chest wall thickness. Acad. Emerg. Med..

[23] Sanchez LD (2011). Anterior versus lateral needle decompression of tension pneumothorax: Comparison by computed tomography chest wall measurement. Acad. Emerg. Med..

[24] Beckett A (2011). Needle decompression for tension pneumothorax in Tactical Combat Casualty Care: Do catheters placed in the midaxillary line kink more often than those in the midclavicular line?. J. Trauma.

[25] Laan DV (2016). Chest wall thickness and decompression failure: A systematic review and meta-analysis comparing anatomic locations in needle thoracostomy. Injury.

[26] Inaba K (2015). Cadaveric comparison of the optimal site for needle decompression of tension pneumothorax by prehospital care providers. J. Trauma Acute Care Surg..

[27] Inaba K (2011). Optimal positioning for emergent needle thoracostomy: A cadaver-based study. J. Trauma.

[28] Moritz S, Kasprzak P, Arlt M, Taeger K, Metz C (2007). Accuracy of cerebral monitoring in detecting cerebral ischemia during carotid endarterectomy: A comparison of transcranial Doppler sonography, near-infrared spectroscopy, stump pressure, and somatosensory evoked potentials. Anesthesiology.

[29] Cope M, Delpy DT (1988). System for long-term measurement of cerebral blood and tissue oxygenation on newborn infants by near infra-red transillumination. Med. Biol. Eng. Comput..

[30] Lin L (2000). Influence of a fat on muscle oxygenation measurement using near-IR spectroscopy: Quantitative analysis based on two-layered phantom experiments and Monte Carlo simulation. Front. Med. Biol. Eng..

[31] Rothbart A (2015). Peripheral intravenous cannulation with support of infrared laser vein viewing system in a pre-operation setting in pediatric patients. BMC Res. Notes.

[32] Boas DA (2001). The accuracy of near infrared spectroscopy and imaging during focal changes in cerebral hemodynamics. Neuroimage.

[33] Baker WB (2014). Modified Beer–Lambert law for blood flow. Biomed. Opt. Express.

[34] Kaspers OP, Sterenborg HJCM, Amelink A (2008). Controlling the optical path length in turbid media using differential path-length spectroscopy: Fiber diameter dependence. Appl. Opt..

[35] Mengelkoch LJ, Martin D, Lawler J (1994). A review of the principles of pulse oximetry and accuracy of pulse oximeter estimates during exercise. Phys. Ther..

